# Zoster Segmental Paralysis: Clinical and Electrodiagnostic Features in a Retrospective Case Series of 17 Patients

**DOI:** 10.7759/cureus.106017

**Published:** 2026-03-28

**Authors:** Lisa B Shields, Vasudeva G Iyer, Christopher B Shields

**Affiliations:** 1 Norton Neuroscience Institute, Norton Healthcare, Louisville, USA; 2 Clinical Neurophysiology, Neurodiagnostic Center of Louisville, Louisville, USA

**Keywords:** electrodiagnostic study, herpes zoster, infectious disease, neurology, zoster segmental paralysis

## Abstract

Background and objective

Herpes zoster infection is due to reactivation of the varicella zoster virus, usually at the dorsal root ganglia, leading to classic skin lesions and neuralgic pain. Concurrent involvement of motor axons/cells may cause segmental paralysis. We sought to describe the clinical and electrodiagnostic (EDX) findings in a cohort of patients with zoster segmental paralysis (ZSP).

Methods

This is a retrospective study of 17 patients with historical and clinical evidence of ZSP of the upper extremities (UE) or lower extremities (LE), confirmed by EDX studies. The clinical metrics collected included (1) demographics; (2) relevant history, including herpes zoster and/or herpes zoster vaccination; and (3) clinical findings (sensory, motor, and reflexes) and localization of the motor deficit. The EDX protocol included motor and sensory nerve conduction studies and needle electromyography (EMG). Metrics collected included motor and sensory conduction velocity, alterations in compound muscle action potentials (CMAP) and sensory nerve action potentials (SNAP), and signs of denervation in muscles detected by needle EMG, as well as their topography for precise localization.

Results

In our study, of the 17 patients with ZSP by history, the UE were involved in 15 (88.2%) patients and the LE in two (11.8%). All patients had weakness of specific muscles of the UE or LE corresponding to the dermatomal location of the skin eruptions. Of the 15 patients who had symptoms of the UE, the most frequent clinical localization of the motor deficit was the C8 and T1 nerve roots (8 (53.3%)). Of the two patients who had symptoms of the LE, the localization of the motor deficit was the L5 and S1 nerve roots in one and the L5 nerve root in the other. Fifteen (88.2%) patients had sensory abnormalities of either the UE or LE, which consisted of either decreased pinprick sensation or allodynia. The EDX studies revealed abnormal motor conduction in 15 (88.2%) patients, displayed by diffuse slowing of motor conduction and/or low amplitude or absence of the CMAP. Sensory conduction was abnormal in 16 (94.1%) patients, with either decreased or absent SNAP. Thirteen (76.5%) patients demonstrated abnormal spontaneous activity suggestive of denervation. Of the 15 patients with UE symptoms, the EDX localization was most frequently at the C8-T1 ventral root/horn cell (V) and dorsal root ganglion (D) in 8 (53.3%) patients. In the two patients with LE symptoms, the EDX location was at the L5-S1 ventral root/horn cell and dorsal root ganglion in one, and the L5 ventral root/horn cell and dorsal root ganglion in the other.

Conclusions

ZSP is a rare complication of herpes zoster infection. Physicians should consider ZSP in the differential diagnosis when patients present with acute onset of muscle weakness in a radicular distribution and should look for skin lesions suggestive of herpes zoster in the corresponding dermatome. EDX studies are highly useful in confirming the radicular/segmental topography of muscle denervation. In this study of a large cohort of patients with ZSP confirmed by EDX, we describe the clinical and EDX findings to improve clinical detection of this uncommon condition.

## Introduction

Varicella zoster virus (VZV) remains dormant in the spinal dorsal root ganglia or cranial nerve ganglia until it reactivates and causes herpes zoster [1]. The elderly and immunocompromised are more vulnerable to herpes zoster, often with pain and a vesicular rash as presenting symptoms [1-4]. Herpes zoster may initiate with pain and pruritus prior to the classic vesicular rash. Approximately 5-20% of patients develop postherpetic neuralgia caused by damage to the sensory ganglia spurred by VZV reactivation and inflammatory changes [1].

In 1972, Thomas and Howard defined zoster segmental paralysis (ZSP) as a rare neurological complication of herpes zoster when the VZV spreads from the dorsal root to the adjacent ventral root, resulting in motor weakness [5]. In their study of 1,210 patients with herpes zoster, 61 (5%) had evidence of ZSP [5]. Between 0.5% and 5% of patients with herpes zoster develop ZSP [1,5]. The segmental weakness usually occurs in the same myotome as the dermatome of the rash [1,2]. The pathogenesis is inflammation caused by the spread of the herpes virus from the dorsal root ganglia to the ventral roots, leading to hypervascularity of the perineural structures or disruption of the blood-nerve barrier [6,7]. The motor impairment may affect the spinal nerve root, plexus, or peripheral nerve. ZSP most commonly affects the head and face [3,8] and upper extremities (UE; primarily in the C5-7 segments) [1,6,7,9-16], although it may involve the lower extremities (LE) [4,7,8,15,17,18], diaphragm [19,20], bladder [21], and abdomen [22,23]. ZSP may manifest as Ramsay Hunt syndrome, with facial nerve paresis and zoster vesicles on the ipsilateral ear pinna [2]. Most of the extant studies of ZSP are case reports or case series. Few larger studies have evaluated this rare condition.

This is a retrospective single-center case series describing the clinical features and EDX localization patterns in patients with suspected ZSP. The differential diagnosis of ZSP and diagnostic techniques to differentiate between various mimics are discussed. The appropriate treatment of ZSP is also presented.

## Materials and methods

Study design and setting

Under an Institutional Review Board-approved protocol, we performed a 14-year (June 2, 2011 to September 29, 2025) retrospective analysis of patients who were referred to our Neurodiagnostic Center for EDX studies in whom the history and clinical examination indicated ZSP. Our American Association of Neuromuscular & Electrodiagnostic Medicine-accredited Neurodiagnostic Center evaluates approximately 1,000 patients annually referred for EDX studies, mainly by hand surgeons and neurosurgeons. All cases evaluated at our Neurodiagnostic Center are labeled with a diagnosis abbreviation for quick data analysis. This feature is unique to our laboratory. The EDX protocol includes nerve conduction and needle electromyography (EMG) studies. We included all patients diagnosed with ZSP based upon clinical evaluation as well as EDX studies. The same electromyographer, board-certified in electrodiagnostic (EDX) medicine and clinical neurophysiology, performed the evaluations of all patients, which negated any inter-examiner reliability bias.

Inclusion and exclusion criteria

Inclusion criteria were patients referred for EDX studies who had a history of herpes zoster and clinical evidence of ZSP. All patients had nerve conduction and needle EMG studies. Exclusion criteria included patients whose EMG/NCV studies revealed entrapment neuropathies. If imaging studies demonstrated compression of the nerve roots at the corresponding level (based on the topography of the EMG abnormalities), these patients were excluded.

Metrics collected

The case definition is an acute onset of muscle weakness following (within days) the occurrence of skin lesions suggestive of herpes zoster. The distribution of muscle weakness and sensory loss is consistent with a radicular lesion. The clinical metrics collected included (1) demographics and laterality (left or right); (2) clinical examination (sensory, motor, and deep tendon reflexes); (3) clinical localization of the motor deficit; and (4) relevant history, including herpes zoster and/or herpes zoster vaccine.

The EDX studies were performed utilizing the standard protocol in our lab [24]. The standard protocol was previously described in detail [24]. The sensory studies were either orthodromic or antidromic [24]. Our lab has standard values with a three-standard deviation value as the normal cutoff. Needle EMG used a monopolar needle, assessing for denervation changes (fibrillations/positive sharp waves as well as alterations in motor unit morphology and recruitment). Muscle weakness and denervation in a radicular distribution and loss of sensory potentials in the same radicular distribution (due to dorsal root ganglion cell loss) were also evaluated. In cervical radiculopathy from other causes, the dorsal root ganglion cells are spared, and, hence, the corresponding sensory nerve action potentials (SNAP) is intact since it is a preganglionic lesion. Muscle weakness from focal neuropathies is excluded, as the muscle involvement is in the nerve rather than the radicular distribution.

The EDX metrics collected included (1) motor conduction (motor conduction velocity and presence of the compound muscle action potentials (CMAP)); (2) sensory conduction (sensory latency and demonstrated features of the SNAP); and (3) needle EMG to document the presence of spontaneous activity such as fibrillations, as well as motor unit potential morphology (duration, amplitude, and polyphasic units) and recruitment pattern during volitional contraction. A sufficient number of muscles were studied to determine the topography of denervated muscles and localize them to single or multiple nerve roots.

Statistical analysis

The descriptive statistics (frequencies and percentages) within this analysis were calculated utilizing Microsoft Excel for Mac, Version 16.107 (Microsoft Corporation, Redmond, WA, USA).

## Results

Demographics

A total of 17 patients had evidence of ZSP on clinical examination (Table 1). The mean age was 71.4 years (range: 52-83 years), and 10 (58.8%) patients were female. ZSP was more common on the right side (11 (64.7%)). It occurred in the UE in 15 (88.2%) patients and the LE in two (11.8%). One patient experienced facial palsy (Ramsay Hunt syndrome) concurrent with weakness of the intrinsic hand muscles. All patients had a history of herpes zoster. One patient received the herpes zoster vaccine six weeks prior to the onset of symptoms. This patient was given the live virus Zostavax in January 2018. The non-live recombinant vaccine Shingrix was approved in October 2017 in the United States, although it was not the exclusive choice until November 2020, when Zostavax was officially discontinued [25]. Two patients in our study had received chemotherapy for malignancies before the appearance of herpes zoster.

**Table 1:** Clinical characteristics of patients with ZSP ADM, abductor digiti minimi; APB, abductor pollicis brevis; EPL, extensor pollicis longus; FDI, first dorsal interosseous; F, female; L, left; M, male; PT, pronator teres; R, right; UE, upper extremities; ZSP, zoster segmental paralysis

Patient no.	Age/sex	Side of symptoms	Physical examination	Clinical localization of motor deficit	Relevant history
1	60/F	R UE	Weakness R intrinsic hand muscles; loss of pinprick sensation R ulnar three fingers	R C8, T1	Two episodes of herpes zoster; R facial palsy (Ramsay Hunt syndrome)
2	83/F	R UE	Weakness of R deltoid and biceps; decreased pinprick sensation R all fingers and palm; R biceps, triceps, and brachioradialis reflexes absent	R C5, C6	Herpes zoster involving R UE; postherpetic neuralgia
3	72/F	R UE	Weakness of R ADM and FDI; decreased pinprick sensation in ulnar two fingers and the ulnar side of the palm	R C8, T1	Herpes zoster R hand; postherpetic neuralgia
4	82/M	L UE	Weakness and wasting L APB, FDI, ADM, and EPL; positive Wartenberg sign	L C8, T1	Herpes zoster over the L palm and medial forearm; postherpetic neuralgia
5	70/M	R UE	Weakness R deltoid, infraspinatus, and biceps; R biceps, triceps, and brachioradialis reflexes absent; decreased pinprick sensation R outer aspect of the upper arm and forearm and radial three digits	R C5, C6	Shingles vaccine six weeks before onset of symptoms; widespread skin lesions, pain in the neck and scapular area, proximal muscle weakness R UE
6	72/M	R UE	Weakness R deltoid and infraspinatus; scars from herpes zoster; decreased sensation R C5 distribution; R biceps reflex diminished	R C5	Herpes zoster in the upper arm; pulmonary malignancy with radiation and chemotherapy
7	52/F	R UE	Weakness R deltoid, biceps, infraspinatus, brachioradialis; loss of sensation R C5 distribution; diminished R biceps reflex	R C5	Herpes zoster in R neck and upper arm three months earlier
8	62/F	R UE	Weakness R intrinsic hand muscles and extensors of fingers; R Wartenberg sign positive; decreased pinprick sensation in all R fingers	R C8, T1	Herpes zoster R hand
9	70/M	L UE	Atrophy of L deltoid and biceps; pinprick sensation decreased over the radial two digits and the radial side of the palm; absent L biceps and brachioradialis reflexes	L C6	Herpes zoster L upper arm; postherpetic neuralgia
10	79/M	L UE	Atrophy and weakness L intrinsic hand muscles; weakness FDP and extensors of fingers; allodynia over ulnar palm and ulnar three digits	L C8, T1	H/O herpes zoster L palm and ulnar two digits; postherpetic neuralgia
11	78/F	L LE	L foot drop; weak extensors and evertors of the left foot	L L5	Herpes zoster L leg
12	72/F	L UE	Weakness L APB and FDI; scars of herpes zoster lesions L C8, T1, and T2 distribution; loss of pinprick over the ulnar aspect of the palm and forearm	L C8, T1	Herpes zoster L hand and forearm; liposarcoma and chemotherapy treatment
13	61/M	R UE	Weakness of R extensors of fingers and intrinsic hand muscles; atrophy of R thenar muscles and 1st dorsal interosseous; decreased pinprick sensation R lateral upper arm, forearm, and all fingers	R C8, T1	Herpes zoster R UE and forearm two months earlier
14	69/F	R UE	Weakness R deltoid, infraspinatus; decreased pinprick sensation R radial aspect of the upper arm	R C5	Herpes zoster R UE and forearm
15	76/F	R LE	Weakness R dorsiflexor of ankle, toe extensors and flexors, inversion of ankle; decreased pinprick sensation dorsum R foot, lateral leg	R L5, S1	Herpes zoster dorsum R foot
16	78/F	L UE	Weakness L APB, FPL, PT; pinprick sensation decreased L radial UE and forearm	L median nerve/C6-8	Herpes zoster L UE and forearm
17	88/M	R UE	Weakness and wasting R APB, FDI; weakness R FPL; allodynia R hand	R C8, T1	Herpes zoster R forearm and palm

Neurological examination

**Figure 1:**
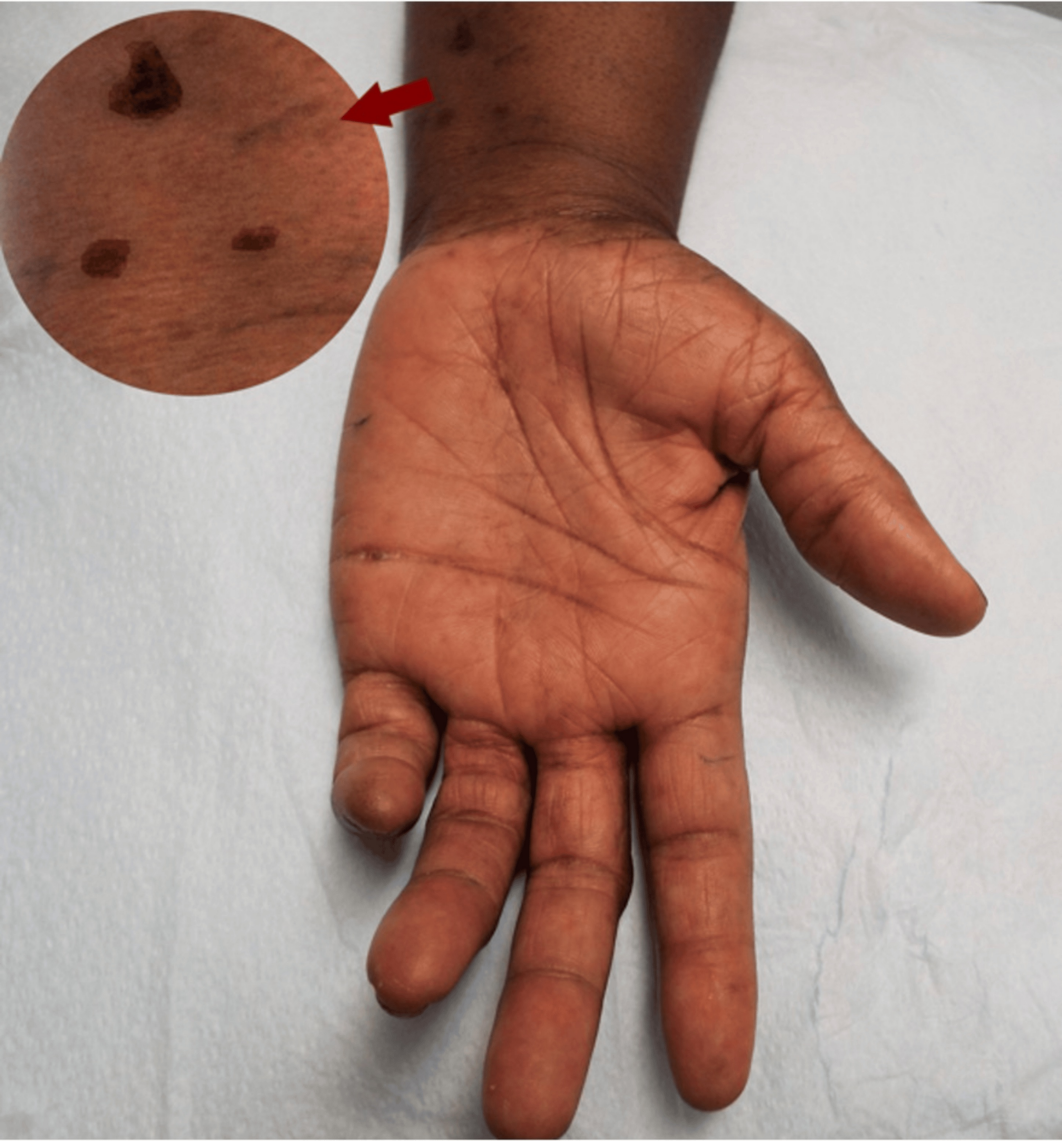
Clawing of the small finger suggestive of C8 radiculopathy

**Figure 2:**
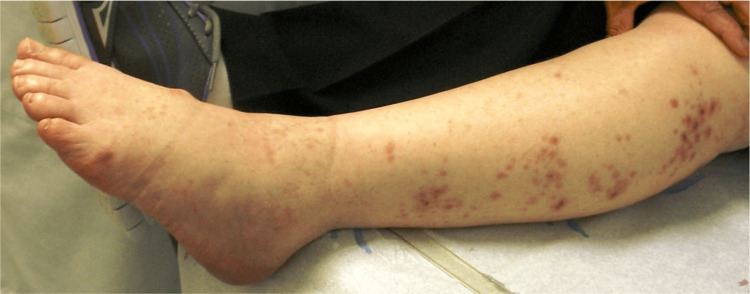
Left foot drop suggestive of L5 radiculopathy and herpetic lesions in the L5 and S1 sensory distribution

**Figure 3:**
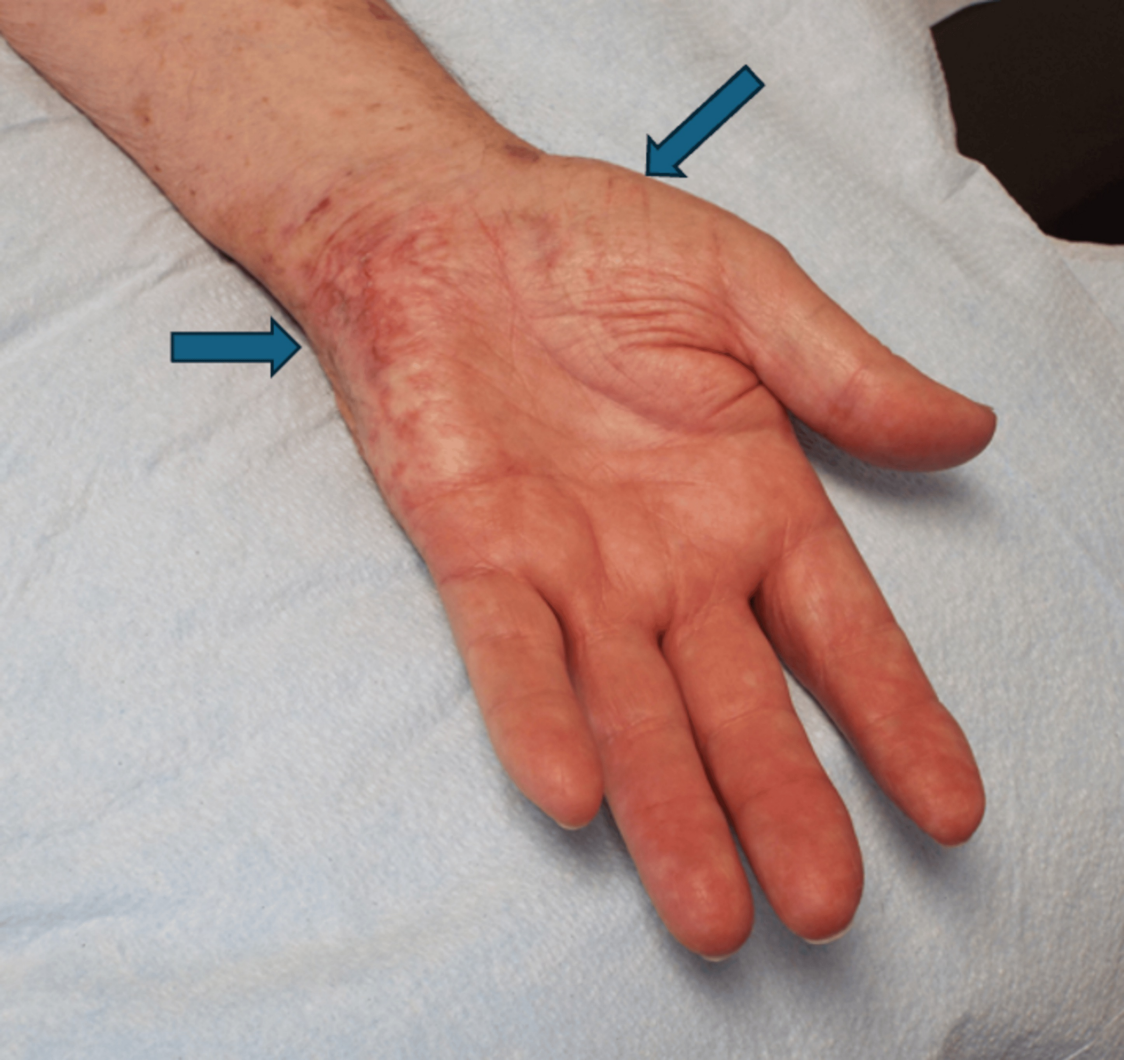
Herpes zoster scars in the C8 dermatome (horizontal arrow) and thenar atrophy suggestive of C8 and T1 myotomes (oblique arrow)

All patients had weakness of specific muscles of the UE or LE corresponding to the dermatomal location of the skin eruptions (Table 2, Figure 1, Figure 2, Figure 3).

**Table 2:** EDX findings of patients with ZSP ADM, abductor digiti minimi; AH, abductor hallucis; APB, abductor pollicis brevis; CMAP, compound muscle action potentials; D, dorsal root ganglion; EDC, extensor digitorum communis; EDX, electrodiagnostic; EI, extensor indicis; EPL, extensor pollicis longus; FCU, flexor carpi ulnaris; FDI, first dorsal interosseous; L, left; MUP, motor unit potentials; OP, opponens pollicis; PT, pronator teres; R, right; SNAP, sensory nerve action potentials; V, ventral root/horn cell; ZSP, zoster segmental paralysis

Patient no.	Abnormal motor conduction	Abnormal sensory conduction	Abnormal spontaneous activity	No MUP or abnormal MUP morphology (MUP duration/amplitude/polyphasic units)	EDX localization
1	None	None	None	R ADM, FDI, APB, EI	R C8, T1 V, D
2	None	Loss of SNAP of the median, ulnar, and superficial radial nerves	None	R deltoid, biceps, triceps, brachioradialis	C5, C6 V; R C6, C7, C8 D
3	Diffuse slowing of motor conduction in the ulnar nerve; low-amplitude CMAP over ADM and APB	Loss of SNAP of digital and dorsal cutaneous branches of ulnar and MABCN; low-amplitude SNAP of the median nerve	R FDI, ADM, FCU	R APB, OP, FDI, ADM, FCU	R C8, T1 V, D
4	Diffuse slowing of motor conduction in the median and ulnar nerves	Loss of SNAP of the ulnar and median nerve branches to the ring finger	L APB, OP	L APB, OP, FDI, ADM, FPL, EPL	L C8, T1 V, D
5	No CMAP over the deltoid on stimulation of the brachial plexus	Loss of SNAP of LABCN; small SNAP over the thumb on median and superficial radial nerve stimulation	R deltoid and infraspinatus	R deltoid, biceps, infraspinatus	R C5, C6 V, D
6	No CMAP over the deltoid on axillary nerve stimulation	LABCN not studied	R deltoid, infraspinatus, rhomboids, biceps	R infraspinatus, deltoid, biceps	R C5, C6 V, D
7	Low-amplitude CMAP over the deltoid and infraspinatus on stimulation of the brachial plexus	LABCN, superficial radial nerve not studied	R deltoid, biceps, brachioradialis, infraspinatus	R deltoid, biceps, brachioradialis, infraspinatus	R C5, C6 V, D not evaluated
8	Diffuse slowing of motor conduction of the R ulnar nerve	Decreased SNAP of the R median and R ulnar nerves	R ADM, FDI, APB, EI	R ADM, FDI, APB, EI	R C8, T1 V, D
9	L axillary and musculocutaneous nerves not evaluated	Loss of SNAP of the L median-thumb digital and superficial radial nerves	None	L deltoid, biceps, triceps	L C6, C7 V, D
10	Low-amplitude CMAP over APB and FDI	Loss of SNAP of the digital and dorsal cutaneous branch of the L ulnar nerve; decreased SNAP of the L median nerve	L FDI, APB, EI, EDC	L FDI, APB, EI	L C8, T1 V, D
11	Diffuse slowing of the L peroneal and tibial nerves	Loss of SNAP of the L superficial peroneal, plantar, and sural nerves	L tibialis anterior and posterior, peroneus longus, tensor fascia lata	L tibialis anterior and posterior, peroneus longus, tensor fascia lata, gastrocnemius	L L5, S1 V, D
12	Diffuse slowing of the L median with small CMAP and the L ulnar with small CMAP	Loss of SNAP in the L median and ulnar nerves	None	L APB, OP, FDI, ADM, EI, FPL	L C8, T1 V, D
13	Diffuse slowing of the R median and ulnar nerves	Loss of SNAP in R median and ulnar nerves	R APB, FDI, EI, EDC	R APB, FDI, EI, EDC	R C8, T1 V, D
14	Axillary and suprascapular nerves not evaluated	LABCN not studied	R deltoid, infraspinatus	R deltoid, infraspinatus	R C5 V, D
15	No CMAP over EDB/tibialis anterior; low-amplitude CMAP over AH	Loss of SNAP in the R superficial peroneal, sural, and plantar nerves	R tibialis anterior and posterior, peroneus longus	R peroneus longus, tibialis anterior and posterior, tensor fascia lata, gluteus medius	R L5 V, D
16	Low-amplitude CMAP over APB	No SNAP over L thumb on stimulation of the median and superficial radial nerve	L FPL	L FPL, PT, FCR, APB, OP	L median nerve/C6-C8 V, C6 D
17	R median nerve: diffuse slowing with low amplitude; CMAP R ulnar nerve: diffuse slowing	Loss of SNAP of R median and ulnar nerves	R FPL	R FPL, APB, OP, FDI, EI	R C8, T1 V, D

Of the 15 patients who had UE symptoms, the most frequent clinical localization of the motor deficit was the C8 and T1 nerve roots (8 (53.3%)), followed by the C5 nerve root (3 (20.0%)), C5 and C6 nerve roots (2 (13.3%)), and the C6 nerve root (1 (6.7%)). Of the two patients who had LE symptoms, the clinical localization of the motor deficit was the L5 and S1 nerve roots in one patient and the L5 nerve root in the other patient. Fifteen (88.2%) patients had sensory abnormalities of either the UE or LE, consisting of either decreased pinprick sensation or allodynia. 

EDX studies

The EDX studies revealed abnormal motor conduction in 15 (88.2%) patients, comprising diffuse slowing of motor conduction and/or low amplitude or absence of the CMAP (Table 2). Sensory conduction was abnormal in 16 (94.1%) patients, with either decreased or absent SNAP. Thirteen (76.5%) patients demonstrated abnormal spontaneous activity. On attempted volitional contraction, the motor unit potentials were either absent or showed abnormal morphology in all patients with respect to duration, amplitude, and polyphasic units.

Of the 15 patients with UE symptoms, the EDX localization was most frequently at the C8-T1 ventral roots (V) and dorsal root ganglion (D) in eight (53.3%) patients; C5-6 V and D in two (13.3%); C6-7 V and D in one (6.7%); C5 V and D in one (6.7%); and C5-6 V with C8-T1 D in one (6.7%). The EDX localizing site was the proximal median nerve/C6 V and C6 D in one (6.7%) patient and C5-6 V (D not performed) in one (6.7%) patient. In the two patients with LE symptoms, the EDX location was at L5-S1 V and D in one patient and L5 V and D in the other patient.

## Discussion

The diagnosis of herpes zoster may be challenging when paralysis occurs prior to the cutaneous lesions, when the myotomal and dermatomal distributions are not concordant, and when MRI or EMG findings are inconsistent with the symptoms [26]. Diagnosis can be even more difficult in zoster sine herpete (herpes zoster without skin lesions) [27]. The initial differential diagnosis may include stroke, compressive spinal myelopathy, meningitis, rotator cuff injury, autoimmune processes, and encephalitis [1,8,26,28]. Patients may undergo cervical surgery when cervical spine pathology and herpes zoster are concurrent [26,28]. One reported patient underwent an anterior cervical discectomy and fusion at C5-6 after a cervical MRI confirmed a disc protrusion at that level [28]. The patient’s symptoms did not resolve postoperatively, and he subsequently developed a herpetic rash. Management with antiviral medications, a nerve root block, and physical therapy resolved his symptoms within three months.

Several diagnostic studies may assist in confirming the etiology of ZSP. A lumbar puncture may be performed to evaluate for CSF pleocytosis, VZV detection by PCR or molecular testing, and immunoglobulins against VZV [4]. Spine MRI may detect hyperintensity in the spinal posterior horn at a vertebral level on T2-weighted images, as well as inflammation of the involved nerve roots and muscle denervation [3,13,14,29]. MRI may also confirm resolution of abnormal imaging findings associated with ZSP when paralysis improves [14]. EDX studies are helpful in confirming ZSP when the cause of monoparesis is uncertain [1]. EDX studies can detect neurogenic lesions and aid in prognosis and estimation of symptom duration [7].

Several studies, mainly consisting of single cases and case series, have utilized EDX studies in the evaluation of ZSP [7,9-12,15-17,20,29]. EDX findings often include reduced amplitude CMAP and SNAP, decreased motor unit recruitment with an increase in polyphasic units, fibrillations, and positive sharp waves in muscles supplied by the affected root [7,16,26,29]. In Aykac et al.’s study of 15 patients with ZSP, all of whom underwent EDX studies, the C8 and T1 nerve roots in the UE and the L5 and S1 nerve roots in the LE were most frequently involved [7]. One patient had a malignancy, and three were treated with immunosuppressants [7]. EDX findings included radicular involvement in three cases, brachial plexus in six, ulnar nerve in one, sciatic nerve in two, and fibular nerve in three. Low-amplitude CMAP and SNAP were detected in 80% of patients, and spontaneous activity on needle EMG was noted in 73% of patients. Axonopathy was the primary pathology in all cases. In Liu et al.’s study of eight patients with ZSP, all of whom had EDX evaluations, the UE was affected in six patients and the LE in two [29]. All patients had evidence of axonopathy. EDX findings included radiculopathy (two patients), plexopathy (two patients), radiculoplexopathy (three patients), and combined radiculopathy and mononeuropathy (one patient). In Kawajiri et al.’s case series of three patients with ZSP, abnormal spontaneous activity was detected in affected muscles by EMG [15].

Our study concurs with the extant literature regarding elderly and immunocompromised patients as the most likely to develop ZSP. One patient received the herpes zoster vaccine prior to developing ZSP. Similar to previous studies, the majority of patients in our study experienced UE symptoms, and EDX studies revealed abnormal motor conduction in most patients, consisting of diffuse slowing of motor conduction and/or low amplitude or absence of the CMAP. Most patients had abnormal sensory conduction and low-amplitude or absent SNAP. Unlike many studies that reported a higher incidence of ZSP at C5-7, the EDX localization in our study agrees with that of Aykac et al. [7], revealing the C8 and T1 nerve roots as the most frequent site of ZSP. We speculate that the predilection for the lower cervical and upper thoracic roots in our study may be due to referral bias, as more patients were sent to our Neurodiagnostic Center by hand surgeons.

While there are no clear guidelines for the clinical management of ZSP, treatment usually involves intravenous acyclovir, gabapentin, and steroids [1,3,7,16]. Early antiviral treatment increases the likelihood of full motor recovery, which usually occurs between six months and one year after symptom onset [1,3].

Strengths and limitations

The strength of the present study is that it features a relatively large number of patients with ZSP and highlights the role of EDX studies in the diagnostic algorithm. The data support descriptive observations rather than strong diagnostic or generalizable claims. Limitations include its retrospective nature and lack of follow-up, as most patients were evaluated only once at our Neurodiagnostic Center, and we were unable to assess the resolution of the patients’ symptoms and signs. Additional limitations include selection bias and limited generalizability.

## Conclusions

ZSP is a rare complication of herpes zoster infection. In this study of a cohort of patients with a history of herpes zoster and clinical evidence of paralysis of UE or LE muscles, we sought to determine the pattern using EDX studies. Physicians should be aware of ZSP when patients present with acute painful motor weakness of the UE or LE. A thorough clinical history, recognition of herpetic lesions, and precise localization using EDX studies can facilitate the diagnosis of ZSP and the timely initiation of treatment.
